# Duplication of 8q24 in Chronic Lymphocytic Leukemia: Cytogenetic and Molecular Biologic Analysis of *MYC* Aberrations

**DOI:** 10.3389/fonc.2022.859618

**Published:** 2022-06-24

**Authors:** Eva Ondroušková, Michaela Bohúnová, Kristýna Závacká, Patrik Čech, Petra Šmuhařová, Miroslav Boudný, Martina Oršulová, Anna Panovská, Lenka Radová, Michael Doubek, Karla Plevová, Marie Jarošová

**Affiliations:** ^1^Department of Internal Medicine – Hematology and Oncology, University Hospital Brno and Faculty of Medicine, Masaryk University, Brno, Czechia; ^2^Center of Molecular Medicine, Central European Institute of Technology, Masaryk University, Brno, Czechia; ^3^Institute of Medical Genetics and Genomics, Faculty of Medicine, Masaryk University, Brno, Czechia

**Keywords:** chronic lymphocytic leukemia, MYC, complex karyotype, 8q24 gain, prognosis

## Abstract

Chronic lymphocytic leukemia (CLL) with cytogenetics findings, such as complex karyotype and deletions of *TP53* or *ATM*, is associated with adverse clinical outcomes. Additional chromosomal abnormalities further stratify patients into groups with diverse prognoses. Gain of 8q24 is one of the abnormalities considered as prognostically unfavorable. In our study, we performed a FISH analysis in an initial cohort of 303 consecutive CLL patients and determined the frequency of +8q to be 6.3 %. Our analysis confirmed the association with *TP53/ATM* aberrations and CK, as the frequency of +8q reached 26.7 % in an extended del*TP53/ATM*+CK cohort. M-FISH analysis enabled the identification of partner chromosomes where the segment of the duplicated 8q arm was localized. More detailed mapping of the gained 8q region using the M-BAND method determined the smallest amplified region 8q23-8qter. We observed significantly shorter overall survival (OS; 9.0 years in +8q-positive vs. 10.6 years in +8q-negative; p=0.02) and detected slightly higher MYC mRNA/protein levels in +8q-positive vs. +8q-negative patients.

## Introduction

Chronic lymphocytic leukemia (CLL) is the most common type of leukemia among adults in the Western world, with a median age of disease presentation of about 70 years. Clinical outcomes for most CLL patients have improved remarkably in the last decade, but there is still a group of high-risk patients whose treatment remains challenging (1). In the era of chemotherapy, these patients progressed in less than two years after initial treatment (2). Independent biomarkers of adverse prognosis include unmutated immunoglobulin heavy chain variable gene (UM-IGHV), *TP53* mutation/deletion, and high complex karyotype (CK), defined as five or more cytogenetic structural/numerical aberrations (3–5). Patients bearing these negative biomarkers benefit from treatment with specific B-cell receptor (BCR) signaling inhibitors and the BCL-2 antagonist in the first line (6).

CLL patients with CK (defined as ≥ 3 cytogenetic aberrations) constitute a heterogeneous group with variable clinical outcomes. It is necessary to study these cases in more detail to reveal subgroups with less favorable prognosis, as shown in a study by Baliakas and colleagues (3). The authors showed that trisomy of chromosomes 12 and 19 predicted an indolent course in patients with CK. On the other hand, in CK with up to four cytogenetic aberrations, the presence of *TP53* aberration predicted an aggressive disease course, similar to the sole presence of high complex karyotype (defined as ≥ 5 cytogenetic aberrations) (3).

According to a study by Leeksma and colleagues, the gain of 8q encompassing the *MYC* gene (+8q) is one of the independent factors significantly associated with shorter overall survival (OS) in CLL patients (7). In the unselected CLL population, a frequency of +8q appears to be low, between 3 – 5 % (8, 9). However, in contrast, in relapsed/refractory cases, +8q is particularly enriched (10). In the context of karyotype complexity, a frequency of +8q is significantly higher in CK than in non-CK karyotypes (11, 12) and often coincides with *TP53* or *ATM* aberrations (13, 14). Nevertheless, the contribution of +8q to adverse outcomes in patients with CK remains unclear (7, 11).

In the tested cohort, we confirmed the association of *TP53/ATM* aberrations and complex karyotype with 8q gain. Additionally, we identified the smallest duplicated 8q region and the partner chromosomes where the duplicated 8q region localizes. Shorter overall survival of +8q-positive patients supported the hypothesis that the 8q gain further contributes to the adverse prognosis of patients with *TP53/ATM* aberrations and complex karyotypes.

## Material and Methods

### Patient Cohorts

In this study, we analyzed peripheral blood samples obtained from CLL patients monitored at the University Hospital Brno, the Czech Republic. For all samples, written informed consent with their research use was obtained in accordance with the Declaration of Helsinki.

In the first part of this study, we performed an initial screening of all consecutive CLL patients examined in our laboratory in 2018 (303 patients in total). This pre-screening aimed to determine the frequency of +8q in unselected CLL population and to identify cytogenetic aberrations that coincide with +8q.

Next, a second patient cohort was selected based on the results of the pre-screening study. All CLL patients tested in our laboratory within the years 2015-2018 who met the condition of CK and del*ATM* (deleted *ATM*) and/or del*TP53* (deleted *TP53*) (90 patients in total) were enrolled for further analysis. The characteristics of the analyzed cohort are in **Table 1**.

**Table 1:** The characteristics of the analyzed cohort of 90 patients with CK.

		Whole dataset		*MYC* pos		*MYC* neg	
Gender	F	31	34%	9	38%	22	33%
	M	59	66%	15	63%	44	67%
Subgroup	del*ATM* + CK	50	56%	7	29%	43	65%
	del*TP53* + CK	28	31%	11	46%	17	26%
	del*TP53* + del*ATM* + CK	12	13%	6	25%	6	9%
OS status	alive	47	52%	11	46%	36	55%
	dead	43	48%	13	54%	30	45%
Rai	I	28	36%	7	32%	21	38%
	II	8	10%	1	5%	7	13%
	III	6	8%	2	9%	4	7%
	IV	13	17%	6	27%	7	13%
	0	23	29%	6	27%	17	30%
Binet	A	44	56%	13	57%	31	56%
	B	15	19%	2	9%	13	24%
	C	19	24%	8	35%	11	20%
IGHV status	MU	10	12%	2	10%	8	13%
	UM	71	86%	18	90%	53	84%
	UM + MU	2	2%	0	0%	2	3%
FISH del(13q) total	Y	70	78%	18	75%	52	79%
	N	20	22%	6	25%	14	21%
FISH del(13q) monoallelic	Y	67	74%	18	75%	49	74%
	N	23	26%	6	25%	17	26%
FISH del(13q) biallelic	Y	17	19%	2	8%	15	19%
	N	73	81%	22	92%	63	81%
FISH del*ATM*	Y	62	69%	13	54%	49	74%
	N	28	31%	11	46%	17	26%
FISH del*TP53*	Y	40	44%	17	71%	23	35%
	N	50	56%	7	29%	43	65%
FISH trisomy 12	Y	5	6%	2	8%	3	5%
	N	85	94%	22	92%	63	95%
*TP53*	MU	45	54%	18	75%	27	45%
	UM	39	46%	6	25%	33	55%
*ATM*	MU	5	71%	0	–	5	71%
	UM	2	29%	0	–	2	29%
Complex karyotype (no of changes)	3 or 4	31	34%	9	38%	22	33%
	5	27	30%	14	58%	13	20%
	ND	32	36%	1	4%	31	47%
*MYC*	pos	24	27%	24	100%	0	0%
	neg	66	73%	0	0%	66	100%
No of treatment lines	median (range)	3 (0-10)		3 (1-10)		3 (0-9)	

F, female; M, male; CK, complex karyotype; OS, overall survival; IGHV, immunoglobulin heavy chain gene; MU, mutated; UM, unmutated; ND, not determined (the exact number of changes in the CK not determined as the detailed analysis by M-FISH method was not performed), Y, yes; N, no.

### Cytogenetic Analysis

Peripheral blood samples were treated according to the stimulation protocol for metaphase induction based on CpG-oligonucleotide DSP30 plus interleukin-2 for 72 hours before fixation and Giemsa staining (15). Karyotypes were captured at magnification 1000x and documented on LUCIA Cytogenetics software (Laboratory Imaging s.r.o, Prague, the Czech Republic). Karyotypes were evaluated according to the recommendations of the ISCN 2020 (International System for Human Cytogenomic Nomenclature). Patients’ karyotypes with 1 (or more) clones with 3 (or more) abnormalities were evaluated as complex karyotypes (CK). A clone had to have at least two metaphases with the same aberration if the aberration was a chromosome gain or a structural rearrangement, and at least three metaphases if the abnormality was a loss of a chromosome (16).

### Molecular Cytogenetic Analyses

For FISH analyses, the probes were hybridized according to the instructions of manufacturers. For detection of del*ATM*, del*TP53*, del(13q) and +12, the standard CLL panel was used (XL ATM/TP53, XL DLEU/LAMP/12cen; MetaSystems GmbH, Altlussheim, Germany). For detection of the *MYC*-coding sequence, probe CL 6q21/8q24 (MetaSystems) was used; this custom-mixed probe is a combination of two locus-specific probes – the 6q21 locus from the XL 6q21/6q23/6cen probe (probe length 304 kb, coordinates D6S1594 – D6S1396E; the results for the 6q21 probe are not elaborated in detail in this study) and the 8q24 locus from the XL MYC amp probe (342 kb, coordinates RH77966 to D8S490). For detection of *MYC* translocations, the break apart *MYC* probe was used (Zyto*Light* SPEC MYC Dual Color Break Apart Probe; ZytoVision GmbH, Bremerhaven, Germany). The proximal part of this probe covers the region approx. 387 kb to 856 kb centromeric of the *MYC* locus including the region of focal gains described by Edelmann and colleagues (8). Hybridization signals in at least 200 nuclei were scored on a Nikon Eclipse Niu fluorescence microscope at magnification 1000x (Nikon Instruments Europe BV, Amsterdam, Netherlands). For the *ATM*, *TP53*, and *MYC*-detecting probes, the threshold for the positivity was set to 10 % to enhance the chance to find metaphases with these aberrations for the M-FISH (multicolor fluorescence *in situ* hybridization) and M-BAND (multicolor banding) analyses. Signals were documented using LUCIA Cytogenetics software (Laboratory Imaging s.r.o, Prague, the Czech Republic).

The routine cytogenetic analysis of G-banded chromosomes allowed the identification of patients with complex karyotypes, but the sensitivity of this method is limited. Therefore, the M-FISH method was used in the cohort of selected patients for a more precise description of all karyotype changes. Subsequently, the M-BAND analysis enabled identifying the extent of the duplicated 8q region with higher accuracy. For M-FISH and M-BAND analyses, probes were hybridized according to the manufacturer’s instructions (24XCyte, XCyte8; MetaSystem). The M-BAND8 probe covered chromosome 8 with different fluorochromes along the entire chromosome length. M-BAND patterns are independent of chromatin condensation and provide a resolution equivalent to the 550-band level for G-bands. The metaphases were captured using an Axio Imager Z2 microscope at magnification 630x (Zeiss, Jena, Germany) and analyzed with the NEON/ISIS software (MetaSystems).

### Gene Expression Analysis Using Quantitative Real-Time PCR

As input material, B lymphocytes separated from peripheral blood using gradient centrifugation on Ficoll-Pague PLUS (GE Healthcare, Uppsala, Sweden) coupled with the RosetteSep^®^ B Cell Enrichment Kit (StemCell Technologies Inc., Vancouver, Canada) were used. RNA was isolated with TRI Reagent (MRC, Cincinnati, USA). As positive controls, RNA samples from cell lines NALM6 and MEC-1 were used. Then, RNA was reverse transcribed into cDNA using SuperScript^®^ II Reverse Transcriptase (Invitrogen, Carlsbad, CA, USA). Gene expression (*APEX1, CDK4, CDKN1A, CDKN1B, CDKN2B, DUSP1, GADD45A, NCL TERT*) was analyzed by real-time PCR on the QuantStudio™ 12K Flex system (both Thermo Fisher Scientific, Waltham, USA) using ThermoFisher Scientific TaqMan assays. The *HPRT1* and *TBP* genes were used as endogenous controls. All reactions were pipetted in triplicates. After removing outlying Ct values (i.e., the values differing from the remaining two replicates by ≥0.3 Ct; 4.6% of Ct values) to correct on the technical accuracy of the method, relative quantification using the 2^-ΔΔCT^ method was performed.

### Antibodies and Immunoblotting

Protein extracts were obtained and subjected to western blot analysis as described previously (17). For MYC and β-actin immunodetection, the following specific primary antibodies were used: MYC (D84C12), β-Actin (13E5) (both Cell Signaling Technology, Danvers, Massachusetts, USA). Secondary antibody: anti-rabbit (7074; Cell Signaling Technology). Chemiluminescence was detected with Clarity™ Western ECL Blotting Substrate (Bio-Rad, Hercules, USA). Signals were quantified with ImageJ Software (www.imagej.net) and referred to the respective controls, i.e., β-actin levels in individual samples. Protein extracts from cell lines NALM6 and MEC-1 were used as positive controls.

### Statistical Analysis

All statistical analyses were performed in freeware R. For statistical comparison of mRNA and protein levels between groups, we used the Kruskal-Wallis ANOVA and the Mann-Whitney test. A logrank test was applied to evaluate differences in survival of distinct groups of patients. The Kaplan-Meier curves were used for visualization of survival in patient groups. Patients after bone marrow transplantation (n=3) were excluded from the OS analysis. The level of significance was set at alpha=0.05.

## Results

### *MYC* Aberrations Are Associated With del*ATM*, del*TP53*, and Complex Karyotypes

**Figure 1:**
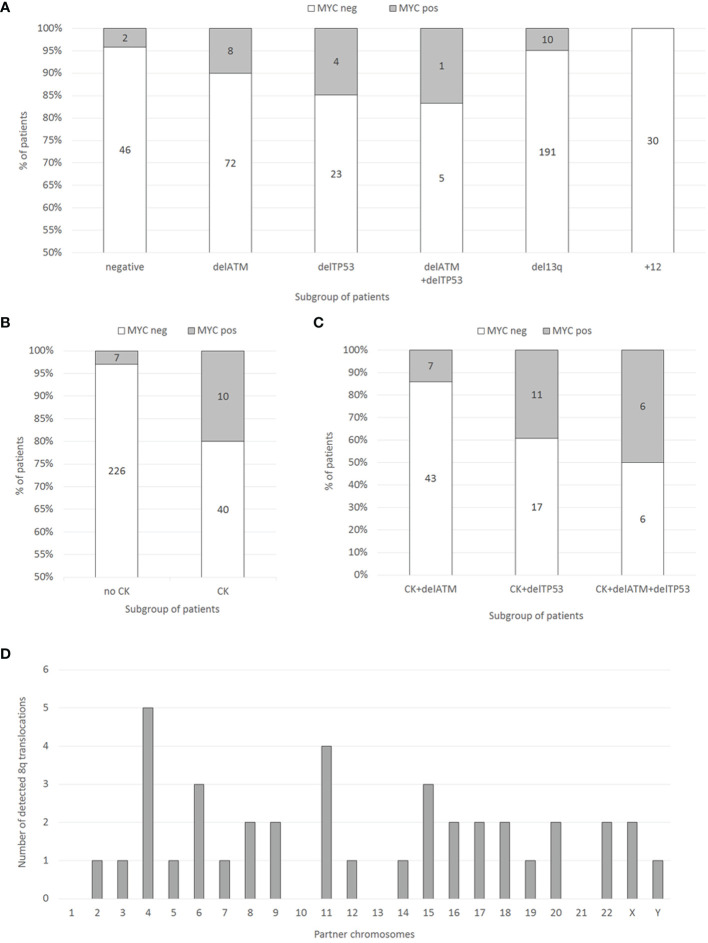
**(A)** Distribution of *MYC* gains in the groups of patients with recurrent aberrations (del*ATM*, del*TP53*, del(13q), +12, or negative) determined by FISH in 303 consecutive CLL patient samples. Numbers within columns represent absolute numbers of patients. **(B)** Distribution of *MYC* gains (determined by FISH) in the groups of patients with or without CK (determined by conventional chromosome banding) in 283 consecutive CLL patient samples. Numbers within columns represent absolute numbers of patients. **(C)** Distribution of *MYC* aberrations (determined by FISH) in 90 patient samples selected for the presence of CK (determined by conventional chromosome banding) together with either del*TP53* or del*ATM*, or both (determined by FISH). Numbers within columns represent absolute numbers of patients. **(D)** The frequency and localization of the duplicated 8q region on individual chromosomes identified by the M-FISH method.

In the pre-screening, consecutive samples from 303 CLL patients (96 treatment-naïve, 194 treated, 13 follow-up loss) were analyzed for DNA copy number changes in our laboratory in 2018 by using the standard diagnostic FISH panel to detect del*ATM*, del*TP53*, del(13q), +12) and by the 6q21/8q24 MetaSystems probes. In this unselected group of patients, the frequency of 8q24 gains covering the *MYC*-coding sequence reached only 6.3 % (19/303). Within the subgroups defined by the most common CLL-related recurrent cytogenetic aberrations, the cases with *MYC* gain reached the following frequencies – del*ATM*: 10.0 % (8/80), del*TP53*: 14.8 % (4/27), del(13q): 5 % (10/201), +12: 0 % (0/30), del*ATM*+del*TP53*: 16.6 % (1/6), and patients negative for the standard CLL-FISH probe panel: 4.2 % (2/48) (**Figure 1A**).

G-banding karyotype analysis (available for 283/303 patients; 93.4% of the cohort) revealed a significant association of *MYC* gain with CK (defined by the presence of ≥3 numerical or structural abnormalities in the same clone). There were 82.3% (233/283) patients without CK, among them 3% (7/233) with *MYC* gain. On the other hand, in the group of patients with CK (17.7% of patients; 50/283), the *MYC* gain was detected in 20 % (10/50; p<0.0001) of cases (**Figure 1B**).

Based on the pre-screening results, we aimed to enrich the cohort with *MYC* aberrations with additional cases tested in our laboratory during the years 2015-2018. Thus, we searched for those meeting the condition of complex karyotype (clone/s with 3 or more cytogenetic aberrations) together with del*ATM*, del*TP53*, or both. The resulting cohort consisted of 90 patients, who had CK together with del*TP53* (28/90, i.e., 31.1 %) or with del*ATM* (50/90, i.e., 55.6 %) or with del*ATM*+del*TP53* (12/90, i.e., 13.3 %) (**Figure 1C**). Basic clinical, cytogenetic, and molecular biologic (IGHV, *TP53*, and *ATM* mutations) characteristics of these 90 patients in the context of *MYC* aberrations are summarized in **Table 1**.

**Figure 2:**
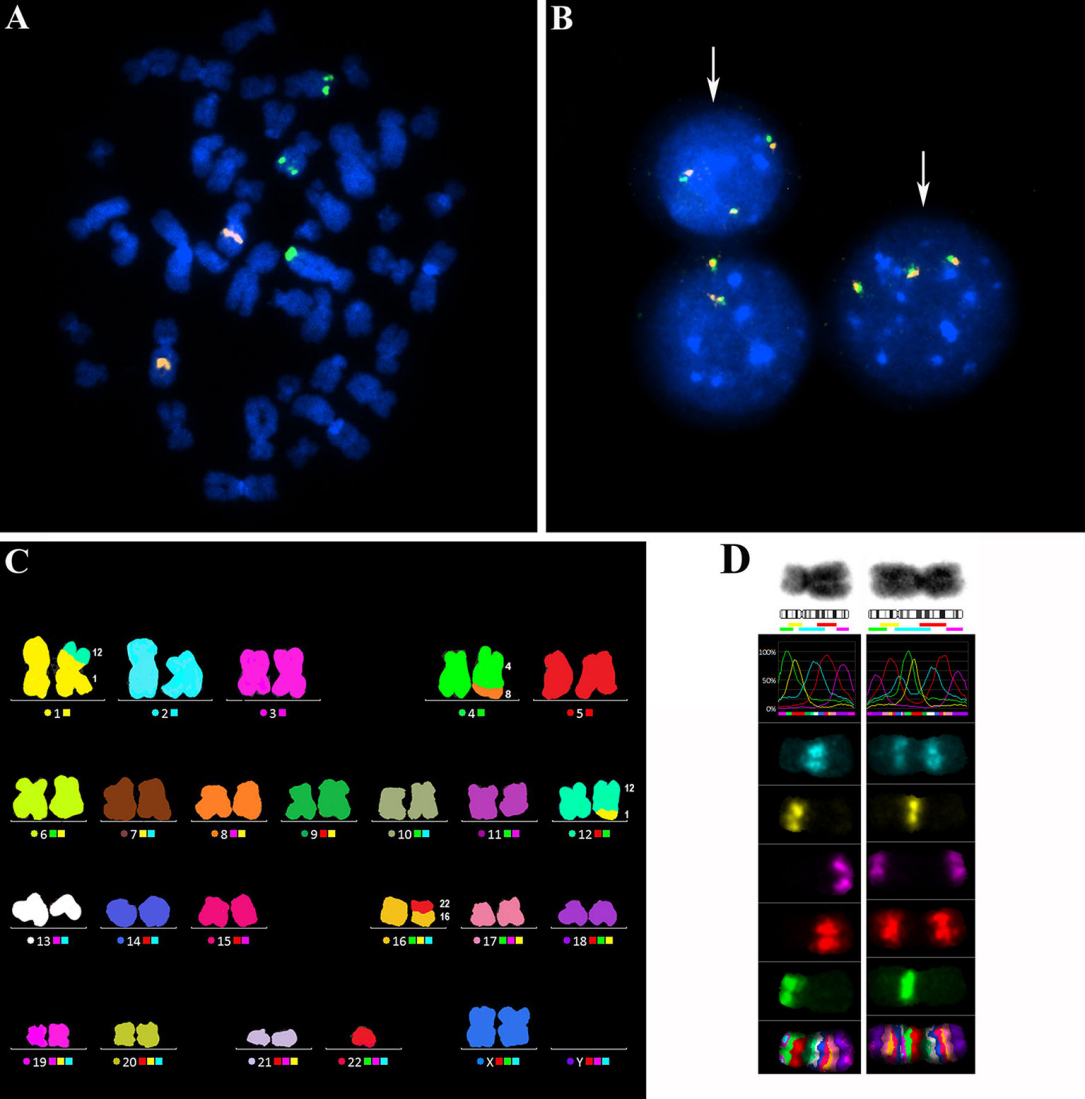
**(A)** A representative metaphasis with three copies of the *MYC* gene visualized by the FISH method. The probe CLL 6q21/8q24 (MetaSystems), covering the *MYC* coding region 8q24 (green signals) and a control region 6q21 (orange signals), was used. **(B)** Representative nuclei with three copies of the *MYC* gene (white arrows), visualized by the FISH method using the *MYC* break-apart probe (ZytoVision). This probe surrounds the common break sites for *MYC* gene translocations. The orange-green fusion signals indicate that the break site is not in proximity to the *MYC* regulatory regions. **(C)** A representative metaphasis hybridized with the M-FISH probe 24XCyte (MetaSystems) for FISH analysis of the whole karyotype. In this metaphasis, one balanced translocation t(1;12) and two unbalanced translocations dic(16;22) and der(4)t(4;8) with 8q gain were detected. **(D)** Analysis of the extent of 8q gain using the M-BAND8 probe XCyte8 (MetaSystems). Normal chromosome 8 (on the left) and a derivative chromosome 8 (on the right) with the duplicated 8q13-8qter region on an 8p-arm.

All 90 additional samples from CLL patients were examined for *MYC* aberrations using FISH i) with the probe covering the *MYC* coding sequence and ii) with the *MYC* break-apart probe. The former probe confirmed a gain of one or more *MYC* gene copies. The latter probe surrounding the common break sites was used to identify breaks in proximity to the *MYC* regulatory regions, i.e., the translocation. Representative FISH results for both probes are shown in **Figure 2** (**Figures 2A, B**). In 24/90 patients (26.7%), *MYC* aberration was detected. *MYC* gain was the predominant change observed in 21/24 (87.5 %) cases as a sole aberration and in 2/24 (8.3 %) cases, it combined with *MYC* translocation. One additional copy of the *MYC* gene (+8q24) was the most common aberration (16/24, i.e., 66.7 %), followed by the combination of two clones with one or two additional *MYC* copies (3/24, i.e., 12.5 %). In one case, two additional *MYC* copies were detected (1/24, i.e., 4.2 %). Interestingly, an extrachromosomal amplification of *MYC* signal (double minutes; dmins) was observed in one patient – this is a rare finding in CLL. The *MYC* translocation was detected in three cases, either as a sole aberration (1 case) or combined with *MYC* gain (2 cases). We also observed that the clone with *MYC* aberration was either smaller or of a similar size as the del*ATM*/del*TP53* clone in most cases (23/24 patients; 95.8 %). All detected types of *MYC* aberrations and the size of clones (% of nuclei) are summarized in **Table 2**.

**Table 2:** Cytogenetic analysis of *MYC* aberrations in 24 *MYC*-positive patients with CK.

Case No	Gender	del*ATM*/del*TP53*/*MYC* aberration clone size (%)	FISH: type of *MYC* aberration	CK (no of changes)	M-FISH/M-BAND: ISCN notation of clone(s) with *MYC* aberration
**1**	F	0/88/70	dmins	≥5	–
**2**	M	0/64/39	3xMYC	≥5	–
**3**	F	0/97/13	3xMYC	3-4	46,XX,der(3)t(3;8)(q?27;**q22.3**),i(17)(q11.2),der(22)t(X;22)(q?13;q13.1)[5]
**4**	M	0/87/76	3xMYC	≥5	44,XY,?inv(3)(p21.2q?27),der(3)t(3;14)(p?21.3);?,del(6)(p21.1p?24),-8,der(10)t(3;10)(?;q?24.3),der(14)t(10;14)(q?;q)?ins(14;17)(q?23);?, der(15)t(8;15)(**q22.1**;q)?,der(15)?del(15)(p12)del(15)(q?13),?dic(17;20)(p?11.2;p?11.2),der(18)t(15;18)(q?;p)?ins(15;8)(q?;**q22.1**)[cp5]
**5**	M	0/91/89	3xMYC	3-4	45,XY,der(6)t(6;17)(q23.1;q?21.3),dic(14;18)(p?11.1;p?11.2),der(20)t(8;20)(**q13**;q?13.3)[12]
**6**	M	0/86/74	3xMYC/4xMYC	3-4	45,XY,dic(13;17)(p?13;p?11.2),der(22)t(8;22)(**q13**;q11.2),der(Y)t(Y;8)(q11.2;**q13**)[8]/45,XY,dic(13;17)(p?13;p?11.2),der(17)t(8;17)(**q13**;q21.3)[2]/45,XY,der(11)t(8;11)(**q21.2**;q?13.5),dic(13;17)(p?13;p?11.2)[1]/45,XY,-3,dic(13;17)(p?13;p?11.2),der(18)t(8;18)(**q13**;q21.1)[1]/45,XY,der(2)t(2;8)(q32.1;**q22.3**),dic(13;17)(p?13;p?11.2)[1]
**7**	F	0/13/15	3xMYC	≥5	76-87,XXXX,-5,-6,der(6)t(6;8)(p?21;**q21.2**),-8,-9,der(9)t(9;15)(?p?;q)?,-10,-13,dic(13;17)(p?11.2;q?11.2),der(14)t(1;14)(?;q?31)x2,-15,+16,der(17)t(17;22)(?p11.2);?,+20,+20,-22[cp11]
**8**	F	0/57/53	3xMYC	≥5	40,XX,-4,-5,der(6),-7,-8,der(12)t(X;12),-15,?i(17q),dic(19;22),der(20)(20pter->?q12::8q24.3->8**q13**::8p11.2->8pter)[2]
**9**	M	0/56/12	3xMYC/4xMYC	≥5	–
**10**	F	0/74/81	3xMYC	3-4	46,XX,der(12)t(8;12)(**q23**;q)?,der(17)t(17;18)(p?12);?,t(11;15)(q?13.1;q?24)[19]
**11**	M	0/75/27	translocation	3-4	46,XY,t(8;22)(**q24**;q?12),der(17)t(2;17)(?;p11.2),t(17;21)(q23.1,q22.1)[4]
**12**	M	17/78/13	4xMYC	≥5	46,XY,der(11)t(8;11)(**q22.1**;q14.1),der(11)t(8;11)(**q22.3**;q22.2),del(17)(p?11.2)[3]/47,X,dic(Y;17)(?p11.2;?p11.2),del(8)(q)?,der(15)invins(15;8)(q?;**q21.3**)[2]/44,X,-Y,?dic(8;14),del(17)(p?11.2)[1]/43,Y,der(X)ins(X;8)(?q13);?,-9,dic(10;15)(?q23.1;p11.2),dic(17;20)(p11.2;?q12)[1]
**13**	M	89/16/67	3xMYC/4xMYC/translocation	≥5	46,XY,del(1)(q?25.1),t(5;10)(q?22;q21.1),t(8;9)(**q24**;q34),t(11;13)(p?11.2;q?14.3),der(17)t(17;17)(q?11.2;q?12)[8]
**14**	M	89/30/46	3xMYC/4xMYC	≥5	–
**15**	F	42/48/27	3xMYC	≥5	–
**16**	M	87/87/56	3xMYC	≥5	46,XY,der(5)t(5;8)(p14;**q13**),?i(17)(q12),der(20)t(2;20)(?;?q12)[15]/45,XY,-8,dup(8)(?q)?,del(17)(p12),der(20)t(2;20)(?;?q12)[4]/43,XY,-7,-8,?dic(8;18)(p11.2;p11.2),del(11)(q?22),?i(17)(q12),der(20)t(2;20)(?;?q12)[3]
**17**	M	4/20/46	3xMYC	3-4	46,XY,?del(1)(?q24q32.2),der(3)t(3;10)(?p25);?,der(15)t(8;15)(?;p?13)[5]/45,Y,der(X)t(X;8)(q?23;**q21**),?dic(13;14)(p11.2;p11.2),der(21)t(13;21)(p13);?[2]/46,XY,?del(1)(p22.1p?35),der(6)t(6;7)(q?21);?,der(16)t(8;16)(**q21**;q?22)[1]/45,XY,der(8)t(4;8)(?;q)?,dic(13;21)(p11.2;p11.2),der(22)t(13;22)(?;p13)[1]
**18**	M	74/0/84	3xMYC/4xMYC/translocation	3-4	46,XY,del(1)(?q21),der(4)t(4;8)(p?14;**q22.3**),der(6)(8qter->**8q23**::6p?21->6q21::6q27->6qter)[3]/46,XY,del(1)(?q21),?del(2)(q?21),der(7)t(7;8)(q?22;**q21.3**)[2]
**19**	M	73/0/20	3xMYC	ND	–
**20**	M	95/0/90	3xMYC	3-4	46,XY,der(4)(4qter->q16::4p14->q12::8**q22.1**->8qter),der(8)(8pter->8p23::8p11.2->8q11.2::4q12->4qter),der(11)t(8;11)(**q13**;q14.1)[24]/46,XY,der(11)t(8;11)(**q13**;q14.1)[2]
**21**	F	96/0/85	3xMYC	3-4	46,XX,t(5;11)(q31.3;q13.3),t(6;10)(p21.1;p11.2),der(22)t(8;22)(**q22.1**;q12.3)[8]/46,XX,t(5;11)(q31.3;q13.3),der(8)(8qter->8**q21.3**::8p23.2->8qter)[3] +der(11)t(8;11)(**q22.1**;q)?[3]
**22**	F	96/0/94	3xMYC	≥5	45,XX,der(9)t(8;9)(**q22.1**;q?32),del(11)(q11.3),-17,der(19)t(17;19)(q?;q13.3)[10]
**23**	M	92/0/67	3xMYC	≥5	46,XY,der(4)t(4;8)(p?14;**q22.1**),del(11)(q14)[3]/45,XY,der(6)t(6;8)(q?21;**q22.1**),der(8)t(8;21)(p)?;?,del(11)(q14),-19[2]/45,X,-Y,t(2;7)(q?24.3;q?32),?der(4)t(4;12)(p)?;?,der(17)t(8;17)(**q23**;q?21.3)[1]/44,XY,der(4)t(4;9)(p14);?,der(9)t(8;9)(**q23**;p?13),del(11)(q14),-16,der(17)t(17;21)(?p)?;?,-21[1]
**24**	F	97/0/41	3xMYC	≥5	45,XX,t(1;12)(p33;q23),der(4)t(4;8)(q33;**q23**),del(11)(q14q)?,?dic(16;22)(?p11.2;p12)[5]/45,XX,t(1;12)(p33;q23),del(11)(q14q)?,del(13)(q14q)?,der(16)t(8;16)(**q23**;q?13)[3] +der(8)(8qter->8**q22.1**::8p23.2->8qter)[2]

F, female; M, male; dmins, double minutes; CK, complex karyotype; M-FISH, multicolor FISH method; M-BAND, multicolor banding method. ND, not determined (the exact number of changes in the CK not determined as the detailed analysis by the M-FISH method was not performed). ISCN, International System for Human Cytogenomic Nomenclature. The position of breaks on chromosome 8 is highlighted in bold.

The *MYC* aberrations identified using the FISH method reached the following frequencies among these CK subgroups – del*ATM*: 14 % (7/50), del*TP53*: 39.3 % (11/28), del*ATM*+del*TP53*: 50 % (6/12). These results were similar to the initial cohort, with the frequency of *MYC* aberrations increasing in del*ATM* -> del*TP53* -> del*ATM*+del*TP53* subgroups. There were no significant differences between the *MYC*-positive and *MYC*-negative groups of patients regarding IGHV mutation status and *ATM* deletion. The *TP53* aberration was detected significantly more frequently (p>0.004) in the *MYC*-positive group: 83.3% (20/24) than in the *MYC*-negative group: 48.5 % (32/66). All but four patients in this cohort were treated previously.

### The Amplified 8q Regions Translocate to Random Chromosomes Within the CK Subgroup

To identify partner chromosomes where the duplicated 8q region was localized, the M-FISH method was performed in 19 of 24 samples with an *MYC* aberration, (in the remaining 5 cases, an insufficient number of metaphases was obtained or no more material was available). In one patient (no. 9), the subclone with *MYC* aberration was not detected in metaphases. The ISCN notation for *MYC*-aberrant clones is summarized in **Table 2**. A representative M-FISH karyotype is shown in **Figure 2** (**Figure 2C**). Our results revealed that the site of integration of the gained 8q region containing the *MYC* gene is random and that there is no recurrent chromosomal partner (**Figure 1D**). Most often, the translocation of the duplicated 8q region was detected on chromosome 4 (5/18 cases; 28%); nevertheless, the localization of the break site differed among the patients; thus, no recurrent target site on chromosome 4 was involved. In 9/18 patients (50 %), the duplicated 8q region translocated to different chromosomal partners in several individual clones (**Table 2**; break sites highlighted in bold). In one patient (no.11), translocation in the *MYC* regulatory region 8q24.21 (as determined using the break-apart probe) without any copy-number change in *MYC*-coding sequences was identified as t(8;22)(q24;q?12). In another patient (no. 18) with both 8q24 gain and translocation detected using the FISH method in interphase nuclei, a minor clone with 8q24.21 translocation was not detected in metaphases.

**Figure 3:**
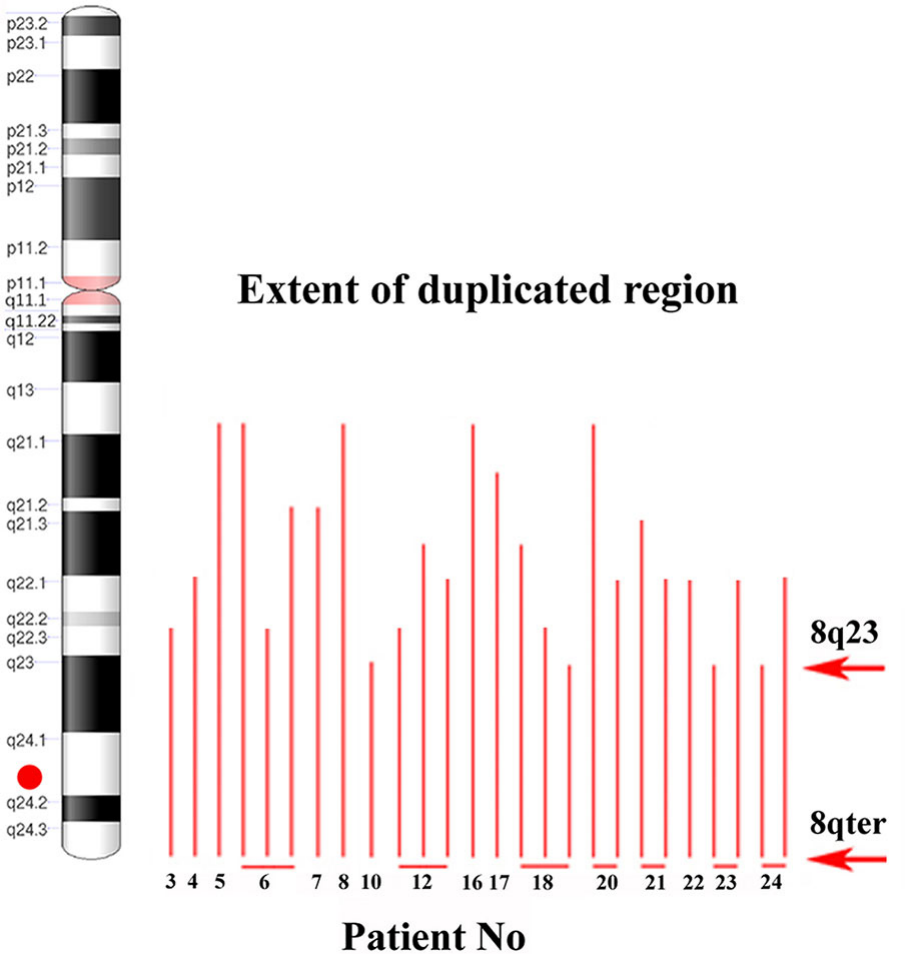
The identification of the smallest duplicated region using the M-BAND method. An ideogram of chromosome 8 on the left side. A red dot indicates the position of the *MYC* gene. Red lines show the size of the duplicated 8q region individual patients (patient numbers under red lines; several lines in one patient indicate more than one clone with *MYC* gain). Red arrows delimit the smallest duplicated region.

To identify the smallest duplicated region and the sites of breaks, the M-BAND8 analysis was performed in 18 of 24 patient samples with *MYC* aberration (in 5 cases, an insufficient number of metaphases was obtained, or no more material was available, in the remaining case no. 9, the subclone with *MYC* aberration was not detected in metaphases). A representative M-BAND8 analysis is shown in **Figure 2** (**Figure 2D**). The results of M-FISH together with the M-BAND8 analysis are summarized in **Table 2**. As shown in **Figure 3**, the duplicated region varied from 8q13-8qter to 8q23-8qter, the latter being determined as the smallest duplicated region in our hands (**Figure 3**).

### Expression of MYC mRNA and Protein Is Slightly Increased in +8q Samples

**Figure 4:**
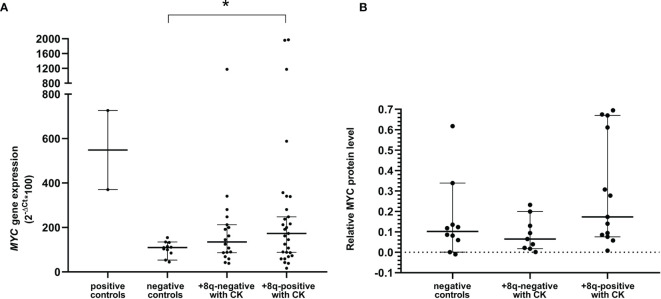
**(A)** Quantification of *MYC* mRNA levels using quantitative real-time PCR (qRT-PCR). **(B)** Quantification of MYC protein levels using western blot immunodetection. Positive controls: cell lines NALM6 and MEC-1. Negative controls: patients negative in both the cytogenetic and molecular-cytogenetic analyses (n=10). +8q-positive with CK; patients with complex karyotype and *MYC* aberration (n=13). +8q-negative; patients with complex karyotype but without *MYC* aberration (n=9). The Mann-Whitney tests were applied to confirm a significant difference in gene expression between the groups. A statistically significant difference (p=0.05) is marked by an asterisk.

Next, we determined the mRNA level of *MYC* and its downstream genes (*APEX1, CDK4, CDKN1A, CDKN1B, CDKN2B, DUSP1, GADD45A, NCL, TERT*) in +8q-positive patients and two control groups, i.e., 10 patients negative in both cytogenetic and molecular-cytogenetic analyses (i.e., 46,XX or 46,XY with CLL-FISH negativity; negative control group) and randomly selected 9 patients with CK and del*ATM*/del*TP53* but without the +8q aberration (+8q-negative CK group). As positive controls, cell lines MEC-1 and NALM6 with a high level of MYC expression were used. No significant difference in the expression of downstream genes was observed between the +8q-positive and +8q-negative groups (**Supplementary Figure 1**). The median values of *MYC* mRNA relative level determined by the 2^-ΔCT^ method were 109.442 in negative controls, 134.9 in +8q-negative CK controls, while 172.969 in +8q-positive CK samples (**Figure 4A**). We also compared the level of MYC protein among the tested groups using western blot immunodetection. Similar to mRNA, we observed higher levels of MYC protein in the +8q-positive CK group when compared to controls (**Figure 4B**). The median values of MYC protein relative level (after normalization to β-Actin) were 0.102 in negative controls, 0.065 in +8q-negative CK controls and 0.173 in +8q-positive CK samples.

### Survival Analysis

**Figure 5:**
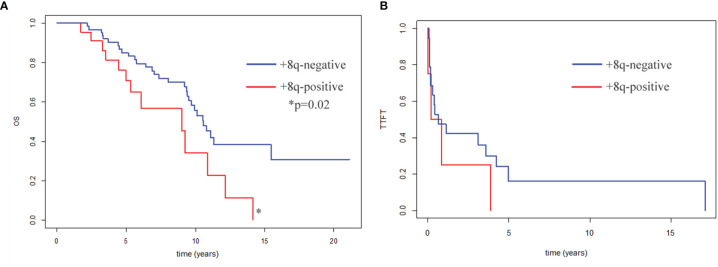
**(A)** Kaplan–Meier curves for patients’ overall survival (OS). OS of +8q-negative patients (n=63) and +8q-positive patients (n=22) was compared. Patients after bone marrow transplantation (n=3) were excluded from the OS analysis. **(B)** Kaplan–Meier plots for time to first treatment (TTFT). TTFT of +8q-negative patients (n=64) and +8q-positive patients (n=24) was compared. Differences were evaluated by a logrank test. A statistically significant difference (p=0.02) is marked by an asterisk.

Eighty-five patients with CK and del*ATM*/del*TP53* were included in the survival analysis (2 patients were excluded due to the follow-up loss, another 3 patients due to bone marrow transplantation). They were divided into two groups, the +8q-negative and +8q-positive groups. We observed significantly shorter median survival for OS in the +8q-positive group (9.0 years in +8q-positive vs. 10.6 years in +8q-negative; hazard ratio 2.14; p=0.02) (**Figure 5A**). No statistically significant difference was observed when comparing the time to first treatment (TTFT) between the +8q-positive vs. +8q-negative patients (**Figure 5B**). The distribution of clinico-biological features of the +8q-negative and +8q-positive groups is summarized in **Table 1**.

## Discussion

CLL exhibits remarkable clinical heterogeneity that often requires the employment of a variety of treatment strategies. Intrinsic (genetics, microenvironment) and extrinsic (therapy) pressures select distinct clones and subclones that can underlie relapsed/refractory disease. Cytogenetically abnormal clones are identified in about 40 – 70 % of newly diagnosed CLL cases by chromosome analysis and about 80% by FISH (18–21). Among chromosomal abnormalities, del*ATM*, del*TP53*, and complex karyotype are associated with poor clinical outcome. Our results are consistent with the findings that duplication of the 8q chromosome arm segment often coincides with both *TP53* and *ATM* aberration and complex karyotype (11–14). We also observed that the +8q clone was either smaller or of a similar size as the del*ATM*/del*TP53* clone in most cases. Furthermore, in both cohorts together, the *MYC* aberration was detected predominantly in patients treated previously (4 treatment naïve vs. 32 treated in *MYC*-positive group/95 treatment naïve vs. 215 treated in *MYC*-negative group). Similar findings were observed in the study by Landau et al. (22). Although the literature suggests that 8q aberration may precede as well as follow the del*TP53* occurrence (23), our results indicate that in most cases, the 8q aberration was gained as a later event in the disease course. Such findings are in concordance with known MYC functions. This protein acts both as a pro-proliferative and pro-apoptotic regulator (24). In cells with damaged apoptotic signaling (*ATM/TP53* aberration), the MYC pro-proliferative effect dominates, and thus a higher level of the MYC protein can provide a selective advantage to cancer cells.

*MYC* expression can be deregulated by mutation, amplification, translocation, regulation of transcription, and RNA/protein stability (25). In CLL cells, the frequency of somatic mutation in the coding sequence of the *MYC* gene is scarce, reaching only 0.4 % according to the COSMIC database of somatic mutations (26). Deregulated *MYC* expression is commonly found in lymphoma due to *MYC*-coding sequence translocation to the vicinity of immunoglobulin enhancers (27). On the other hand, the +8q aberration that we describe in our group of patients presumably adds one copy of the *MYC* gene while preserving the intact regulatory and coding sequences. Edelmann and colleagues described two types of gains, broad gains covering the *MYC* locus and focal gains (<500 kb) in the super-enhancer region (8). We did not detect these focal gains in the super-enhancer region (the range of our smallest duplicated region was 8q23-8qter), although the FISH probe we used covered its locus (the proximal part of the break apart *MYC* probe). It supports the finding that only the broad +8q gains are enriched in high-risk CLL cases, while the focal gains are relatively rare (1.4 %) independently of risk groups (10).

Importantly, in CLL patients with high MYC mRNA/protein levels, a significantly shorter time to first treatment was observed, showing that *MYC* might be one of the negative prognostic factors (28). The presence of one or more additional copies of the *MYC* gene should, in theory, lead to a higher *MYC* expression. We detected slightly higher levels of MYC mRNA/protein in +8q-positive vs. +8q-negative patient samples, nevertheless, high variability and a small number of samples precluded obtaining statistically significant results. Physiologically, the *MYC* expression is strongly induced by activating stimuli in germinal centers of lymph nodes (LN) and its activation has a localized and transient nature (29). Likewise, Herishanu and colleagues showed that the MYC mRNA/protein level is high in CLL cells isolated from the LN compared to relatively low MYC mRNA/protein levels in the quiescent cells circulating in peripheral blood (30). The remarkable feature of the MYC mRNA/protein is its very short half-life (30 min/20 min) (31). Therefore, we suppose that the MYC level rapidly decreases after leaving the lymph node, and in peripheral blood, only residual mRNA/protein levels are detected. Together, this reasoning might explain why we failed in finding any correlation between the level of mRNA/protein expression and the size of the clone with *MYC* aberration. On the other hand, other mechanisms (mentioned above) deregulating the expression and especially the stability of MYC mRNA/protein can explain higher MYC levels in patients without the 8q gain.

Localization of the translocating break site exactly to 8q24 (within the *MYC*-regulatory region) usually leads to deregulation of *MYC* expression due to the proximity of strong transcription enhancers (typically immunoglobulin’s; IGH, IGK, IGL) without changing the number of *MYC* coding sequences. In CLL, about two-thirds of reported cases with *MYC* translocations involved immunoglobulin partners, while in the remaining cases, less common breakpoints with an unknown effect on *MYC* expression were observed, as reviewed in the study of Fonseka and Tirado (32). *MYC* translocation, either with immunoglobulin genes or other unknown partners is one of the changes acquired in about 16 % - 37 % of CLL patients with Richter’s transformation (33–36). On the other hand, translocations with a gain of 8q have not been mapped in detail yet. Here we describe that the distribution of +8q to other chromosomes is rather random, though the most common translocation partner was chromosome 4 (5/18 cases). Interestingly, we also detected the gained 8q region on the p-arm of one of the chromosomes 8 in 2/18 patients. Generally, the *MYC* gain might be challenging to detect in karyotype with the routinely used G-banding method. Without FISH analysis, this aberration often remains cryptic, especially in subclones, and without M-FISH analysis, the partner chromosomes remain largely unmapped.

Regarding the clinical impact of +8q, the genomic array-based study of the largest cohort so far (2293 cases) revealed that the 8q gain encompassing the *MYC* gene is an important factor significantly associated with shorter OS (7). The *MYC*-affected downstream pathways include the B cell receptor signaling (37), which implies a possible interference with Bruton tyrosine kinase inhibitors and conceivably challenging treatment of CLL patients with *MYC* abnormalities. Indeed, *MYC* upregulation correlated with ibrutinib resistance in mantle cell lymphoma cell lines (38). In contrast, another study (101 cases) did not prove a significant independent clinical impact of *MYC* aberrations (11). In our cohort, the *MYC* aberrations were significantly associated with *TP53* aberrations. In a retrospective study investigating 195 cases with del*TP53*, the 8q24 gain was a significant predictor of short OS in multivariate analysis (39). In concordance with these assumptions, we observed significantly shorter OS in patients with +8q in our cohort of patients. As reviewed by Nguyen-Khac, the double-hit CLL (bearing *TP53* aberration + *MYC* gain) might have an inferior outcome even within the del*TP53* group, but these results from a limited retrospective study have yet to be confirmed in larger cohorts of patients (23). In the study of Leeksma et al., *MYC* gain correlated with UM-IGHV and higher karyotype complexity, another two important factors contributing to unfavorable prognosis (7). In our cohort of patients with CK, the distribution of cases with UM-IGHV did not differ between the +8q-positive and +8q-negative groups. On the other hand, *MYC* aberration correlated with higher karyotype complexity within our dataset. *MYC* deregulation promotes an overall induction of chromosomal instability, as reviewed in several studies (40, 41). Therefore, we conclude that 8q24 gain together with del*TP53* and complex karyotype have a synergistic impact on outcome and predict a particularly poor prognosis. Larger studies are warranted to fully understand the role of *MYC* in the context of other negative biomarkers and its impact on the outcome of high-risk CLL patients.

## Data Availability Statement

The raw data supporting the conclusions of this article will be made available by the authors, without undue reservation.

## Ethics Statement

Ethical review and approval was not required for the study on human participants in accordance with the local legislation and institutional requirements. The patients/participants provided their written informed consent to participate in this study.

## Author Contributions

EO and MBOH performed the FISH, M-FISH and M-BAND analysis, PŠ performed the cytogenetic analysis. KZ with KP (RNA) and MBOU with PČ (proteins) carried out the expression experiments. LR performed the statistical calculations. AP, MO and MD were responsible for the clinical data. EO wrote the manuscript with support from MJ and KP. MJ supervised the project, contributed to the interpretation of the results and to the final manuscript. All authors contributed to the article and approved the submitted version.

## Funding

Supported by the program for the conceptual development of research organization (FNBr 65269705), MUNI/A/1330/2021, and AZV project NU21-08-00237 provided by the Ministry of Health, the Czech Republic.

## Conflict of Interest

MD: Honoraria and Research grants from Roche, AbbVie, AOP Orphan, Astra Zeneca, Gilead and Janssen-Cilag. AP: Honoraria and Travel grants from Roche, Gilead and Janssen-Cilag.

The remaining authors declare that the research was conducted in the absence of any commercial or financial relationships that could be construed as a potential conflict of interest.

## Publisher’s Note

All claims expressed in this article are solely those of the authors and do not necessarily represent those of their affiliated organizations, or those of the publisher, the editors and the reviewers. Any product that may be evaluated in this article, or claim that may be made by its manufacturer, is not guaranteed or endorsed by the publisher.

## Supplementary Material

Supplementary Quantification of mRNA levels of MYC-downstream genes using quantitative real-time PCR (qRT-PCR). Positive controls; cell lines NALM6 and MEC-1. Negative controls; patients negative in both the cytogenetic and molecular-cytogenetic analyses (n=10). +8q-positive with CK; patients with complex karyotype and MYC aberration (n=13). +8q-negative; patients with complex karyotype but without MYC aberration (n=9). No significant difference among individual groups was detected (the pairwise U-test, Bonferroni corrected, was used to calculate the p-value). The median values of relative expression are stated down in the table.
