# Supplementary figures and images for: Duplication of 8q24 in Chronic Lymphocytic Leukemia: Cytogenetic and Molecular Biologic Analysis of MYC Aberrations
Source: Front Oncol. 2022 Jun 24;12:859618. doi: 10.3389/fonc.2022.859618 (PMC9263084; doi:10.3389/fonc.2022.859618)

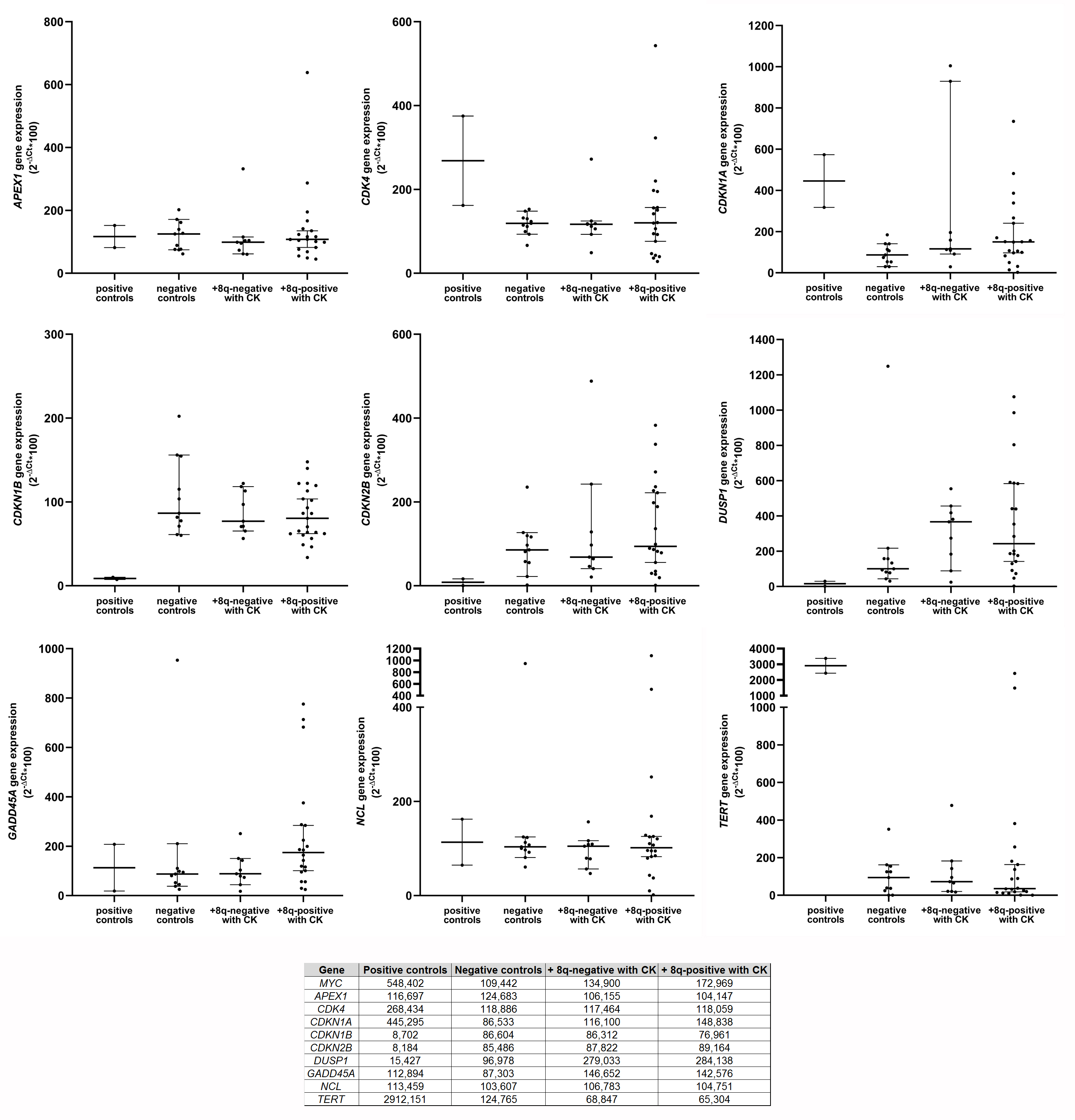

Supplement: Supplementary — Quantification of mRNA levels of MYC-downstream genes using quantitative real-time PCR (qRT-PCR). Positive controls; cell lines NALM6 and MEC-1. Negative controls; patients negative in both the cytogenetic and molecular-cytogenetic analyses (n=10). +8q-positive with CK; patients with complex karyotype and MYC aberration (n=13). +8q-negative; patients with complex karyotype but without MYC aberration (n=9). No significant difference among individual groups was detected (the pairwise U-test, Bonferroni corrected, was used to calculate the p-value). The median values of relative expression are stated down in the table. [file Image_1.jpeg]
